# Treatment of pelvic fractures through a less invasive ilioinguinal approach combined with a minimally invasive posterior approach

**DOI:** 10.1186/s12891-015-0635-x

**Published:** 2015-07-25

**Authors:** Lei Zhu, Lu Wang, Di Shen, Tian-wen Ye, Liang-yu Zhao, Ai-min Chen

**Affiliations:** Department of Orthopedic Trauma Surgery, Changzheng Hospital, The Second Military Medical University, 415 Fengyang Rd., Huangpu District, Shanghai, China; Department of Orthopedic Surgery, Zhoushan Hospital, Zhejiang, China

**Keywords:** Pelvic fractures, Minimally invasive approach, Ilioinguinal approach

## Abstract

**Background:**

Unstable pelvic fractures usually result from high-energy trauma. There are several treatment modalities available. The purpose of this study was to evaluate the clinical application of a new less invasive ilioinguinal approach combined with a minimally invasive posterior approach technique in patients with unstable pelvic fractures. We also address the feasibility, validity, and limitations of the technique.

**Methods:**

Thirty-seven patients with unstable pelvic fractures were treated with our minimally invasive technique. The anterior pelvic ring fractures were treated with a less invasive ilioinguinal approach, and the sacral fractures were treated with a minimally invasive posterior approach. The clinical outcome was measured using the Majeed scoring system, and the quality of fracture reduction was evaluated. The patients were followed up for 13 to 60 months (mean, 24 months).

**Results:**

Anatomical or near to anatomical reduction was achieved in 26 (70.3 %) of the anterior pelvic ring fractures and a satisfactory result was obtained in another 11(29.7 %). For the posterior sacral fractures, excellent reduction was obtained in 33 (89.2 %) of the fractures, with a residual deformity in the other 4 patients. One superficial wound infection and two deep vein thromboses occurred, all of which resolved with conservative treatment. The clinical outcome at one year was “excellent” in 29 patients and “good” in 8 patients (Majeed score).

**Conclusions:**

The satisfactory results showed that a reduction and fixation of unstable pelvic fractures is possible through a combination of a limited ilioinguinal approach and posterior pelvic ring fixation. We believe our method is a new and effective alternative in the management of pelvic fractures.

## Background

Unstable pelvic fractures usually result from high-energy accidents [1]. Patients who sustain these injuries often have concomitant life-threatening conditions, such as haemorrhage and associated neurological and visceral injuries [2]. Hence, pelvic fractures are associated with high mortality and morbidity rates. Successful management of an unstable pelvic fracture is a challenge for all involved disciplines including the orthopaedic surgeon. Several treatment modalities are available for the management of unstable pelvic injuries. The use of external fixation can restore a distorted pelvic ring to a roughly normal configuration. Its use is limited, however, because of its inaccurate reduction and poor ability of the frames to stablize unstable pelvic ring fracture [3]. Surgical exposure for open reduction and fixation provide direct visualisation of the injured pelvic areas. Internal fixation has been shown to be biomechanically superior to external fixation [4, 5]. However, additional blood losses and disruption of early tamponade and clot formation should not be ignored.

For fractures of the anterior pelvic ring, which were generally treated using an ilioinguinal approach, Stoppa approach, or percutaneous intramedullary fixation [6], we recommend a similar but less invasive anterior approach for treating pubic ramus fractures. We hypothesised that complications caused by surgical dissection would be reduced when using this approach. Furthermore, the technique is not so demanding as percutaneous fixation.

The use of iliosacral screws to stablize posterior pelvic ring fractures was described by Matta and Saucedo [7]. They used closed reduction techniques to realign pelvic ring disruption. The blood losses and operation time are both minimal. Recognised complications such as the possibility of nerve root injuries, reduction and fixation failure, superior gluteal artery injury, however, are not uncommon. [8–10] In this study, we used the pedicle screw system for posterior pelvic ring fractures. The less invasive ilioinguinal approach combined with a minimally invasive posterior approach is presented in a series of 37 consecutive patients, who had sustained pelvic fractures.

## Methods

### Preoperative evaluation

**Fig. 1:**
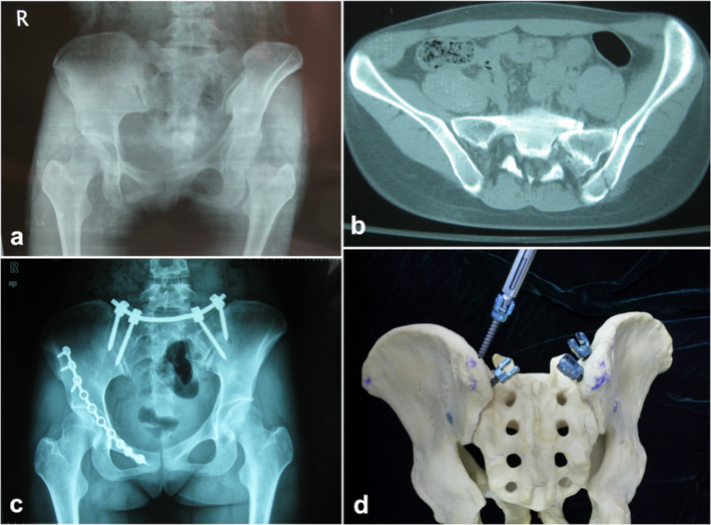
Patient with closed unstable pelvic ring disruption. A 43-year-old woman had a type B (Tile classification) pelvic fracture caused by a traffic accident. **a** Preoperative pelvic radiographic image. **b** Preoperative computed tomographic image. **c** Postoperative pelvic radiographic image. **d** Pelvic model demonstrates two pedicle screws in each dorsal iliac crest

A total of 37 patients with closed, unstable pelvic ring disruptions were treated from January 2008 and September 2012 at our department. All patients were evaluated based on anteroposterior, inlet and outlet plain pelvic radiography (Fig. 1a) and computed tomography scans of the pelvis (Fig. 1b). Patients were excluded from the study if they (1) had pubic symphysis separation, (2) bilateral sacral fracture, (3) sacroiliac joint injuries, (4) sacral plexus injuries that required neurolysis or decompression, or (5) injuries of unclear date or a history of conservatively or surgically treated pelvic injuries. Specific sacral fractures such as comminuted fractures, sacral dimorphism, sacral fractures with inadequate intra-operative images, which are difficult to be treated by percutaneous screws, are the best indications for this technique. Based on the rotational and vertical instabilities of the patients’ injuries, their pelvic fractures were classified as Type B or Type C according to the Tile classification [11] . There were 9 B1 cases, 17 B2 cases, 9 C1 cases and 2 C2 cases (Table 1). All patients underwent reduction and internal fixation of a pelvic fracture using our less invasive anterior approach, combined with a minimally invasive pedicle screw system for a posterior pelvic ring fracture. Written informed consent for participation in the study was obtained from all patients. The research was in compliance with the Helsinki Declaration. The medical ethics committee of the Second Military Medical University gave ethical approval (reference number 2007–029).

**Table 1:** Patients’ characteristics

Sex	Age (years)	Type of fracture	Cause of fracture	Concomitant injuries	Time from injury to surgery (days)	Operation time (min)	Blood loss (ml)	Reduction for fracture (anterior)	Reduction for fracture (posterior)
Male	41	Tile B2	Fall injury	No	1	160	200	Anatomic	Anatomic
Male	38	Tile B1	Traffic injury	Right humerus fracture	4	100	180	Near to Anatomic	Anatomic
Male	35	Tile B2	Fall injury	No	3	180	200	Anatomic	Anatomic
Female	43	Tile C1	Traffic injury	Left femoral neck fracture	4	140	150	Near to Anatomic	Anatomic
Male	36	Tile B2	Fall injury	No	4	100	200	Satisfactory	Anatomic
Female	34	Tile B1	Traffic injury	Left and right alcaneal fractures	5	110	100	Near to Anatomic	Anatomic
Male	46	Tile C1	Fall injury	Left calcaneal fracture	4	150	200	Satisfactory	Anatomic
Female	34	Tile C1	Traffic injury	No	5	110	100	Near to Anatomic	Anatomic
Male	40	Tile B2	Traffic injury	Left olecranon fracture	3	140	200	Anatomic	Anatomic
Male	26	Tile B2	Fall injury	Left calcaneal fracture	4	150	150	Satisfactory	Anatomic
Male	43	Tile B2	Fall injury	No	3	95	90	Near to Anatomic	Anatomic
Male	47	Tile B1	Fall injury	No	5	85	100	Anatomic	Anatomic
Female	33	Tile B2	Crush injury	No	4	100	90	Anatomic	Posterior translation and rotation
Male	46	Tile B2	Crush injury	Left humerus fracture, Multiple rib fractures,Dislocation of right hip joint	14	110	140	Satisfactory	Anatomic
Female	44	Tile C1	Crush injury	No	7	100	150	Anatomic	Anatomic
Female	20	Tile C1	Traffic injury	Mutiple rib fractures, Right olecranon fracture, Right femoral neck fracture	13	90	80	Anatomic	Anatomic
Female	25	Tile B2	Fall injury	Left distal radius fracture	6	90	95	Anatomic	Anatomic
Male	33	Tile B2	Fall injury	No	5	80	100	Satisfactory	Anatomic
Male	40	Tile B1	Crush injury	Right intertrochanteric fracture	5	70	90	Satisfactory	Anatomic
Female	42	Tile C2	Traffic injury	Right acetabular fracture	1	85	120	Satisfactory	Anatomic
Male	40	Tile B2	Crush injury	L1 compression fracture	3	80	90	Anatomic	Anatomic
Male	38	Tile B1	Crush injury	No	4	115	95	Anatomic	Anatomic
Female	46	Tile C1	Crush injury	No	6	95	80	Near to Anatomic	Anatomic
Female	43	Tile C1	Fall injury	Left and right calcaneal fractures	7	90	100	Near to Anatomic	Anatomic
Male	38	Tile C2	Crush injury	Right acetabular fracture	3	100	80	Satisfactory	Anatomic
Female	42	Tile B1	Traffic injury	No	2	85	90	Anatomic	Anatomic
Female	43	Tile B2	Fall injury	Right calcaneal fracture	4	80	90	Anatomic	Anatomic
Male	41	Tile C1	Crush injury	No	4	110	130	Anatomic	Anatomic
Male	39	Tile B2	Traffic injury	No	4	120	130	Anatomic	Posterior translation and rotation
Female	37	Tile B2	Crush injury	Left and right distal radius fractures	3	70	80	Anatomic	Anatomic
Male	36	Tile B1	Traffic injury	No	6	105	135	Satisfactory	Anatomic
Male	47	Tile B2	Traffic injury	Rib fracture, Liver laceration, Intestinal laceration	3	110	150	Near to Anatomic	Posterior translation and rotation
Female	22	Tile B1	Fall injury	No	4	100	180	Anatomic	Anatomic
Female	57	Tile B1	Traffic injury	Multiple rib fractures	7	200	210	Satisfactory	Anatomic
Female	30	Tile B2	Traffic injury	Meniscus tear of right knee	4	90	110	Near to Anatomic	Near to Anatomic
Male	34	Tile B2	Fall injury	Transverse process of lumbar vertebra fracture(L4、5)	5	180	250	Near to Anatomic	Posterior translation and rotation
Male	58	Tile C1	Traffic injury	No	5	200	240	Anatomic	Anatomic

The study group included 21 men and 16 women. The average age was 40.9 years (range, 20–58 years). There were 10 crush injuries, 13 falls from a height, and 14 traffic accidents. All patients were evaluated in the emergency room as soon as they reached our hospital. Initial assessment revealed that three patients were hemodynamically unstable, so their vital signs were stabilised emergently. Surgery for the other patients was scheduled as early as possible to obtain anatomical reduction. The three patients who required immediate vital sign stabilisation needed a relatively longer time after resuscitation in the intensive care unit before undergoing surgery. All patients provided written informed consent before the operation. The mean time from injury to operation was 4.7 days (range, 1–14 days).

### Operative technique

#### Less invasive ilioinguinal approach for anterior ring fracture

The patient was first positioned supine on a radiolucent operation table. Surgical preparation included the abdomen and pelvic region. A catheter was inserted into the bladder before operation.

**Fig. 2:**
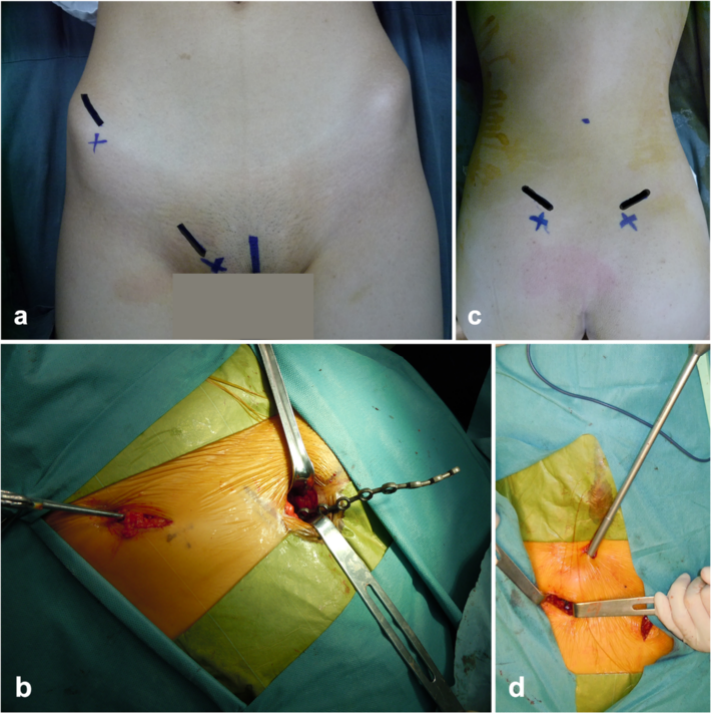
Operative steps. **a** Marking for the incision for exposure through the lateral and medial window. **b** A preflexed reconstruction plate is manoeuvred from the lateral window to the medial window. **c** Marking for the posterosuperior iliac spine and iliac crest. **d** Tightening the screws with a wrench through a small incision 8 cm proximal to the midpoint between the bilateral posterosuperior iliac spines

The incision for exposure through a lateral window extended along the anterior one-third of the iliac crest and ended at the anterosuperior iliac spine (Fig. 2a marked with a thick black line in the area of the anterosuperior iliac spine). Because the lateral femoral cutaneous nerve exits into the thigh over a distance approximately 2 cm medial to the anterosuperior iliac spine, the incision ensures that the lateral femoral cutaneous nerve is protected. The exposure progresses to the iliac crest using sharp dissection. The abdominal muscles and start of iliacus muscle are sharply incised from their origins. After subperiosteal elevation, the iliacus muscle is dissected from the internal iliac fossa to the anterior inferior iliac spine.

For exposure through a medial window, a 3 to 4 cm incision is made along the line from the pubic tubercle to the pubic ramus (Fig. 2a). The incision is then extended to the inferior border of the inguinal ligament and continued through the subcutaneous tissue. After separating the deep fascia underneath the inguinal ligament, the spermatic cord (in men) or round ligament of the uterus (in women) was retracted and protected. The fascia around the iliopectineal and pectineal muscle was separated to expose the superior ramus of the pubis. The iliopsoas and neurovascular bundle could be elevated with the help of a blunt periosteal detacher, providing access to the prepared lateral window. After reduction by traction and reduction clamps, (and, if necessary, temporary fixation with Kirschner wires), a preflexed reconstruction plate with 10–12 holes was slid from the lateral window (Fig. 2b), underneath the neurovascular bundle and iliopsoas muscle, to the medial window. After adjusting its position with fluoroscopic guidance, the plate was fixed on the pelvis with two or three screws at both end of the steel plate.

#### Polyaxial pedicle screws for posterior pelvic fixation

After the anterior ring fracture was fixed, the anteroposterior, inlet and outlet plain radiographs were obtained introperatively using a C-arm machine to examine the posterior pelvic ring. The patient was positioned prone to fix the posterior ring fracture with polyaxial pedicle screws. The surgeon had marked the posterosuperior iliac spine and iliac crest before the operation (Fig. 2c marked with a thick black line in the area of the posterosuperior iliac spine). After disinfecting and draping the area, bilateral incisions 3 cm long were made along the posterosuperior iliac spine. The drill point was located at the first third of the dorsal iliac crest in sagittal section and 1 cm medial to the posterosuperior iliac spines in coronal section. We used a bone awl to open and widen the cortical bone. To determine the trajectory for the screw and create its tract in the ilium, the pedicle probe is angled 15°from the vertical line in coronal section. In the sagittal plane, an angle of 30°to 40° was preferred to minimise discomfort and soft tissue irritation. After two 50-mm-long and 6.5-mm-diameter polyaxial pedicle screws (LegacyC System, Medtronic sofamor, USA) were inserted into the dorsal iliac crests, a 5.5-mm-diameter precontured bar (LegacyC System, Medtronic sofamor, USA) was manoeuvred subcutaneously with the help of a vessel clamp and, connected with the two screws. The reduction and screw positions were checked intraoperatively using anteroposterior, inlet, outlet and LC2 plain radiographs. A small incision was made 8 cm proximal to the midpoint between the bilateral posterosuperior iliac spines for tightening the screws with a wrench (Fig. 2d). In five patients with comminuted fracture of the sacrum, we inserted two pedicle screws in each dorsal iliac crest, the second drill point was located at the iliac crest and 2 cm inferior to the posterosuperior iliac spines (Fig. 1c, d).

### Postoperative treatment and follow-up

After surgery, all patients received an antibiotic against possible infection for one to two days and low-molecular-weight heparin for 1 week to avoid deep venous thrombosis during the hospitalisation. Postoperatively, all the patients were instructed to stay in bed and participate in lower limb and joint functional exercise for 4 weeks and then walk with partial weight bear for 6 weeks. Finally, they were allowed to walk normally 12 weeks after surgery. Complications were defined as infections, deep vein thrombosis, nerve and vascular injuries, erosion of soft tissues overlying the screw head, sexual or urinary dysfunctions and nonunions. The follow-up visits were arranged at 6 weeks, 12 weeks, 24 weeks, 1 year and 2 years postoperatively for clinical and radiographic examinations. The postoperative reduction was evaluated according to the anteroposterior, inlet and outlet pelvic views. Clinical outcome was measured using a scoring system described by Majeed [12]. Five criteria were chosen for functional assessment after major pelvic fractures: pain, standing, sitting, sexual intercourse and performance at work.

## Results

The mean operation time was 112.0 min (range 70–200 min). Estimated blood loss was 131.3 mL (range 80–250 mL). The average hospital stay was 8 days (range 4–18 days). There were no obvious intraoperative injuries to blood vessels, nerves, or other viscera. The quality of anterior pelvic ring fracture reduction was expressed as anatomic (0–1 mm displacement), satisfactory (2–3 mm displacement), or unsatisfactory (>3 mm displacement) according to the criteria of Matta [13]. Anatomical reduction was achieved for 26 (70.3 %) anterior pelvic ring fractures and a satisfactory result was obtained for another 11 (29.7 %) fractures. For posterior fractures, the radiographic results were graded by the maximal residual displacement in the posterior injury to the pelvic ring. The reduction was excellent when anteriorposterior translation or vertical displacement was <5 mm [14]. Excellent reduction was obtained for 33(89.2 %) sacral fractures, although, the other four patients had residual deformity which was >5 mm. One patient who had multiple injuries developed a superficial wound infection at the incision to expose the lateral window during his hospital course. It was treated conservatively with antibiotics and had resolved at the 6–week–follow-up. Two patients developed a deep vein thrombosis before the operation. After receiving anticoagulant as a routine prophylactic measure, both were treated successfully. The patients were prospectively followed up for 13 to 60 months (mean, 24 months) in our outpatient department. No sexual or urinary dysfunction or other complications occurred during the follow-up period. The clinical outcome at 1 year was “excellent” in 29 patients and “good” in 8 patients (Table 2).

**Table 2:** Majeed scores of 37 patients at the last follow-up (12 months)

No.	Pain	Work	Sitting	Sexual intercourse	Standing	Total	Grade
1	25	16	8	4	32	85	Excellent
2	25	20	8	4	32	89	Excellent
3	25	20	8	4	30	87	Excellent
4	20	16	6	4	28	74	Good
5	25	20	10	4	34	93	Excellent
6	20	16	8	4	34	82	Good
7	25	20	8	4	32	89	Excellent
8	25	20	10	4	34	93	Excellent
9	20	16	6	4	28	74	Good
10	25	20	10	4	32	91	Excellent
11	30	16	10	4	36	96	Excellent
12	25	20	10	4	30	89	Excellent
13	30	20	10	4	36	100	Excellent
14	25	12	10	4	30	81	Good
15	25	16	8	4	36	89	Excellent
16	30	16	10	4	36	96	Excellent
17	30	20	10	4	36	100	Excellent
18	30	20	10	4	30	94	Excellent
19	30	12	10	4	36	92	Excellent
20	25	16	8	4	30	83	Good
21	25	20	10	4	36	95	Excellent
22	30	16	8	4	36	94	Excellent
23	30	20	10	4	36	100	Excellent
24	25	16	10	4	32	87	Excellent
25	30	20	10	4	36	100	Excellent
26	30	20	8	4	34	96	Excellent
27	30	16	8	4	36	94	Excellent
28	25	20	10	4	36	95	Excellent
29	30	20	10	4	34	98	Excellent
30	25	20	10	4	36	95	Excellent
31	30	20	10	4	34	98	Excellent
32	25	12	8	3	32	80	Good
33	30	20	8	4	36	98	Excellent
34	30	16	10	4	36	96	Excellent
35	25	16	8	3	30	82	Good
36	25	12	8	3	34	82	Good
37	30	16	10	4	36	96	Excellent

## Discussion

In this study, we used limited anterior and posterior approaches to treat a series of 37 patients who presented with an unstable pelvic ring fracture. No complications such as intraoperative injuries to vessels or nerves, sexual or urinary dysfunction, nonunions occurred. Satisfactory clinical and radiological outcomes were gained. There were 2 patients in our series with deep venous thrombosis occurring preoperatively but no further thromboembolic event in the postoperative phase. One patient developed a superficial wound infection at the incision and was treated with antibiotics successfully.

High-energy pelvic fractures are severe lesions that are associated with significant mortality and morbidity rates. Because of the large force required to disrupt the pelvis, pelvic fractures indicate that the patient has experienced high-energy trauma, and therefore often combined with concomitant injury [15]. Historically, pelvic fractures had been treated using a variety of methods depending on their severity. There is wide consensus that conservative treatment by traction or pelvic sling confines the patient to long bed rest with a high risk of potential complications. The application of external fixation for pelvic fractures reduces bleeding and helps manage hemodynamically unstable patients. It fails, however, to ensure sufficient stability for unstable injuries [16, 17]. Other drawbacks to external fixation include hardware prominence, fixator loosening and reoperations [18–20]. There has been an increasing interest in the use of internal and stable fixation such as plates, sacral bars, and iliosacral screws for treating unstable pelvic fractures. There is also a growing body of evidence that early operative reduction and fixation of pelvic fractures contribute to lower morbidity and mortality rates and shorter hospital stays [3, 21]. The classic anterior approach to the pelvis is the ilioinguinal approach, which provides total and complete access to the anterior column from the sacroiliac joint to the pubic symphysis [22]. Use of the ilioinguinal approach for complex pelvic fractures appears to be associated with a high rate of good to excellent results. However, potential complications with this approach involve mainly the lateral cutaneous nerve of the thigh, neurovascular injuries, and asymmetric expansion of the abdomen when coughing [23]. Zobrist et al. [24] addressed these problems with the help of an endoscope. Their endoscopic technique facilitated reliable internal fixation of anterior ring fractures with minimal soft tissue trauma. This technique, however, is technically demanding, time-consuming and needs a special instrument. Vaidya et al. [25], in a recent study that included 24 patients, presented a novel internal fixation device for stabilising unstable pelvic fractures using supra-acetabular spinal pedicle screws and a subcutaneous connecting rod. There were no infections, delayed unions, or nonunions, and all fractures healed without significant loss of reduction. The authors did, however, report neuropraxia in two patients, and one patient required repositioning of the pedicle screw and readjustment of the screw rod, which had caused discomfort. Their incisions were directly over the anteroinferior iliac spine for pedicle screw placement, which is a high-risk zone in regard to injuring the lateral femoral cutaneous nerve. A similar study by Heisterman et al. [26] was a randomized controlled trial that compared anterior pelvic external fixation versus anterior pelvic internal fixation for unstable pelvic ring injuries. They presented the idea of an anterior pelvic bridge, which is a percutaneous method for fixing the anterior pelvis through limited incisions over the iliac crest and pubic symphysis. In addition to the inherent limits of minimally invasive pelvic fixation (e.g., the lack of direct visualisation), their method required adequate posterior ring stability. Thus, it could be used only in patients who have residual instability anteriorly after the posterior pelvis has been verified to be stable. Yu [27] et al. introduced a similar minimally invasive plate osteosynthesis (MIPO) technique for the treatment of pubic ramus fractures in 15 patients. For exposure of the medial window, the ligaments and the pyramidal muscle were partially excised in their study, which may cause hernias because of the abdominal exposure. In contrast, neither abdominal muscle nor inguinal ligament was involved in the process of exposing the medial window in our study. We experienced no postoperative pulmonary embolism or other thromboembolic event in this series. The incidence of embolism was lower than that described in the literature. Although we recruited only 37 patients for this study, which is too low to make any definitive conclusions, we believe our approach did not result in a high incidence of thromboembolic events. No sexual or urinary dysfunction or other complications occurred during the follow-up period, which was in line with the results of others who used the anterior approach [23]. For posterior pelvic fractures, Matta and Saucedo [7] evaluated the results of three treatment techniques for unstable pelvic fractures in 1989, and introduced a technique for fixation of the posterior pelvic fractures using iliosacral screws. Routt et al. [28] described a method for percutaneous iliosacral screw fixation that caused less soft-tissue violation and blood loss, which is particularly useful in patients with multiple trauma. Because of the complexity of the pelvic anatomy, sacral variation, and the limitations of fluoroscopic control, however, there are certain recognised complications, such as screw misplacement, neurological impairment, and malreduction [29]. In an anatomical cadaver study, Collinge et al. [8] found that the deep superior branch of the superior gluteal nerve and vessels are at significant risk during percutaneous placement of iliosacral screws even when “well placed”. The injury rate in their study was 18 %. In our study, two pedicle screws connected by a precontoured bar were used to stabilize the posterior pelvic ring, a technique similar to that introduced by Sar and Kilicoglu [30]. The biomechanical studies reported in their study showed satisfactory results. The posterior pelvic fixation in the current study was not so solid as the iliosacral screw fixation or lumbopelvic fixation. As a result, the patients were encouraged to stay in bed and participate in functional exercise for 4 weeks, which is long when compared to the literature.

In our study, the anterior approach consisted of a lateral window for the ilioinguinal approach and a medial window. With less blood loss and fewer complications, it was sufficient to achieve anatomical reduction and stable fixation for unstable pelvic fractures. It was not so technically demanding as percutaneous fixation. In addition, it was less invasive as compared with the ilioinguinal approach. The present study has several limitations. First, we used an analysis based on clinical cases to predict the stability of the fixation. To obtain validated results, a rigorously designed case–control study is needed. Second, the study lacks direct biomechanical evidence to support the stability of the fixation. Third, the small number of cases mean that the final evaluation of this technique needs further investigation. Fourth, the reduction proved difficult compared to that with the classic ilioinguinal approach. One case had to be converted to the ilioinguinal approach because of the difficulty of using reduction forceps.

We are encouraged by the satisfactory efficacy provided by the minimally invasive technique in the present study. However, more cases must be investigated and analysed to see whether the clinical outcome can be improved and the complication rate can be lowered as compared with what has been reported in published literature. We believe that our method is an effective alternative for managing pelvic fractures and can be recommended to treat such injuries in the future.

## Conclusions

Pelvic fractures are challenging injuries to manage. Early internal fixation and stablization of unstable pelvic ring injuries can decrease morbidity and improve long-term results. We are encouraged by our results, which showed that reduction and fixation of unstable pelvic fractures is possible using a combination of a limited ilioinguinal approach and a minimally invasive posterior approach. Although a lager number of pelvic fractures must be treated by our method to confirm that the complication rate is truly lowered and the prognosis is improved compared with other operative techniques, we believe that our method is a new and effective alternative for managing pelvic fractures.
